# Unveiling the Genetic Footprint: Exploring Somatic Mutations in Peripheral Arterial Disease Progression

**DOI:** 10.3390/biomedicines11082288

**Published:** 2023-08-17

**Authors:** Amankeldi A. Salybekov, Mehdi Hassanpour

**Affiliations:** Qazaq Institute of Innovative Medicine, Regenerative Medicine Division, Cell and Gene Therapy Department, Astana 010000, Kazakhstan

**Keywords:** peripheral arterial diseases, somatic mutation, thrombosis, atherosclerosis, inflammation, genetic disorders

## Abstract

Peripheral arterial diseases (PADs) are complex cardiovascular conditions influenced by environmental factors and somatic mutations in multiple genes involved in hematopoiesis and inflammation. While traditional risk factors, such as smoking, hypercholesterolemia, and hypertension, have been extensively studied, the role of somatic mutations in PAD progression remains underexplored. The present article intends to provide a comprehensive commentary of the molecular mechanisms, genetic landscape, prognostic significance, and clinical implications of somatic mutations in PADs. The expansion of clonal hematopoiesis of indeterminate potential (CHIP) clones in the circulating blood, named clonal hematopoiesis (CH), leads to the infiltration of these clones into atherosclerotic plaques and the production of inflammatory cytokines, increasing the risk of cardiovascular diseases, including PADs. Furthermore, recent experimental evidence has demonstrated the involvement of somatically mutated TP53 genes with a high variant allele frequency (VAF) in PAD development and prognosis. This review delves into the relationship between CH and PADs, elucidating the prevalence, impact, and underlying mechanisms of this association. This understanding paves the way for novel therapeutic approaches targeting CHIP to promote tissue regeneration and improve outcomes in PAD patients. It emphasizes the need for further research to fully unravel the genetic footprint of the disease and highlights potential clinical implications. The findings presented in this article lay the foundation for personalized medicine approaches and open avenues for the development of targeted therapies based on somatic mutation profiling.

## 1. Introduction

Peripheral arterial diseases (PADs), which encompass a cluster of conditions attributed to a narrowing or occlusion of the arteries supplying blood to the lower extremities, result in reduced blood flow, claudication, non-healing ulcers, and, in severe cases, tissue necrosis or gangrene, posing significant morbidity and mortality risks that make them a global healthcare burden [1]. The complex interplay of genetic and environmental factors contributes to the development and progression of PADs [2]. Thus, understanding the underlying impaired mechanisms and pathophysiology of this condition is crucial for early detection, appropriate management, and the development of novel therapeutic approaches.

**Figure 1:**
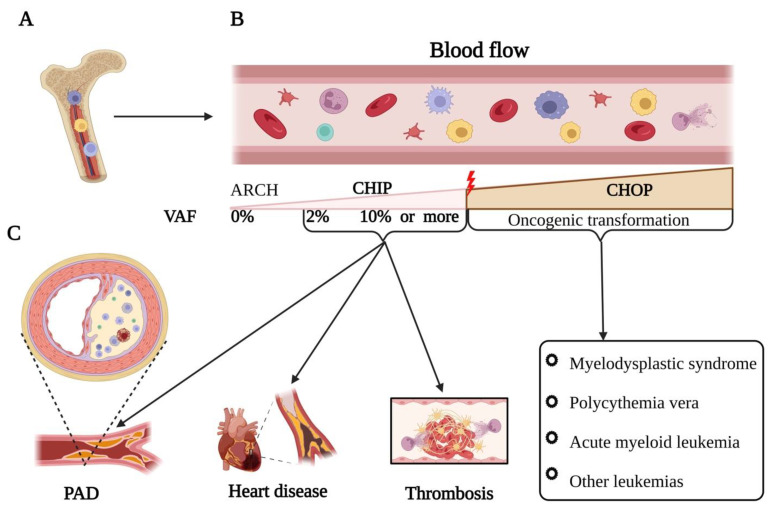
CHIP mutation and its impact on the cardiovascular system: (**A**) Clonal hematopoiesis (CH) occurs when somatic mutations in hematopoietic stem cells (HSCs) start producing the same clone of cells, leading to the expansion of mutant blood cells. (**B**) It is well-documented that the somatic mutation with a variant allele frequency (VAF) less than 2% are known as age-related clonal hematopoiesis (ARCH). However, clonal hematopoiesis of indeterminate potential (CHIP) is defined as the presence of myeloid cancer-associated somatic mutations with a VAF of ≥2% in the hematopoietic cells of individuals without hematologic malignancy or it may transform into an oncogenic status, also known as clonal hematopoiesis with oncogenic potential (CHOP). (**C**) CHIP-mutated cells infiltrate into the atherosclerotic plaque and secrete enormous pro-inflammatory cytokines, such as IL-1β, IL-6, etc., initiating immune-thrombosis and occlusion of the vessel.

Somatic mutations are genetic alterations that occur in the DNA of non-germ cells, including the cells of the arterial walls in PADs. These mutations can arise from a variety of factors, including exposure to environmental toxins, chronic inflammation, and DNA replication errors [3]. The relevance of somatic mutations in PAD progression lies in the potential to disrupt critical cellular processes involved in maintaining vascular health, promoting atherosclerotic plaque formation, altering the vascular tone, and remodeling and modulating angiogenesis [2]. Somatic mutations can affect genes involved in lipid metabolism, extracellular matrix (ECM) remodeling, and endothelial cell (EC) function, thereby influencing plaque stability and arterial remodeling [4]. Additionally, mutations in genes regulating angiogenesis can impact the development of collateral vessels, which are essential compensatory mechanisms in PAD [5]. For example, mutations in genes that regulate vascular smooth muscle cell (VSMC) function, EC integrity, and inflammation can influence the development and progression of atherosclerosis [6]. These mutations may alter the behavior of vascular cells, leading to increased cell proliferation, impaired cell death (apoptosis), enhanced inflammation, and aberrant responses to oxidative stress [7]. Furthermore, somatic mutations can disrupt lipid metabolism, affecting the stability and function of genes involved in this process, which in turn plays a crucial role in atherosclerotic plaque formation. Disruptions in lipid metabolism can result in the accumulation of cholesterol and other lipids within the arterial walls, further exacerbating the occlusion of the arteries in PAD and may contribute to the pathogenesis of the disease (Figure 1) [8].

First of all, there are several genetic terms in this field that are deemed to be defined: As mentioned above, a somatic mutation is a genetic alteration that occurs in non-germline cells during a person’s lifetime, rather than being inherited from their parents. Of note, clonal hematopoiesis (CH) denotes the proliferation of a solitary mutated lineage of blood cells, usually in the bone marrow (BM), and the incidence of a dominant population of blood cells with a specific somatic mutation. Aging-related clonal hematopoiesis (ARCH) represents non-harmful CH characterized by the existence of acquired genetic mutations in the blood or BM, whose frequency escalates with advancing age, with a variant allele frequency (VAF) below 2% [9]. Also, clonal hematopoiesis of indeterminate potential (CHIP) refers to a condition marked by the existence of CH without any concurrent hematologic malignancy [10]. The current diagnostic criteria for CHIP encompass the following: (1) the lack of evident hematological disorders, (2) a typical blood count within normal parameters, and (3) the presence of mutated cells carrying notable driver mutations in at least 2% of white blood cells found in the peripheral blood, as signified by VAF [11]. CHIP is often linked to an elevated risk of cardiovascular disease (CVD) and other age-related conditions [12]. Furthermore, clonal hematopoiesis of oncogenic potential (CHOP) refers to CH occurring in a clinical setting where there is a notable probability of evolving into an overt malignancy [9]. Moreover, VAF points out the proportion of sequencing reads that contain a specific variant allele compared to the total number of reads at that genomic position, which is used to estimate the abundance of a particular mutation within a sample [13] (Graphical Abstract).

Understanding the role of somatic mutations in PAD is important for several reasons. Firstly, it helps to elucidate the underlying molecular mechanisms involved in disease development, providing insights into potential therapeutic targets. By identifying specific mutations associated with PADs, researchers can develop personalized treatment approaches tailored to individual patients’ genetic profiles. Secondly, the detection of somatic mutations in PAD patients may serve as a diagnostic tool for assessing disease severity and progression, enabling clinicians to monitor patients more effectively and adjust treatment strategies accordingly.

## 2. Molecular Mechanisms of Somatic Mutations in PAD

The etiology of somatic mutations in PADs is multifactorial. Several risk factors, including aging, exposure to environmental toxins, chronic inflammation, radiation, and oxidative stress, can induce DNA damage and the subsequent accumulation of somatic mutations [14]. Additionally, genetic predisposition and underlying vascular diseases, such as atherosclerosis, can increase the susceptibility to somatic mutations in PAD. Epidemiological studies have provided valuable insights into the prevalence and distribution of somatic mutations in PAD. Large-scale genomic sequencing efforts have identified specific somatic mutations in genes associated with vascular biology, inflammation, and DNA repair pathways [13]. CHIP-PAD and CHIP pan-arterial atherosclerosis mutations most frequently occur in genes responsible for regulating epigenetics (DNA methyltransferase 3 alpha (DNMT3A) and Tet methylcytosine dioxygenase 2 (TET2)), DNA damage repair (DDR) genes (protein phosphatase 1D (PPM1D), tumor protein p53 (TP53) and breast cancer 1/2 (BRCA1/2)), cell cycle and transcriptional regulator genes (janus kinase 2 gene (JAK2), and additional sex combs like-1 (ASXL1)) [15], as well as mutations specifically disrupting splicing factor genes (putative RNA-binding protein Luc7-like 2 (LUC7L2), pre-mRNA-processing splicing factor 8 (PRPF8), splicing factor 3b subunit 1 (SF3B1), serine- and arginine-rich splicing factor 2 (SRSF2), U2 small nuclear RNA auxiliary factor 1 (U2AF1), and zinc finger CCCH-type, RNA-binding motif and serine/arginine-rich 2 (ZRSR2)) [16]. These studies have revealed the heterogeneity of somatic mutations across PAD patients, highlighting the importance of understanding their impact on vascular biology. Various types of somatic mutations can occur in PADs, each with distinct effects on vascular biology. Point mutations, insertions, deletions, and structural rearrangements can disrupt critical genes involved in vascular homeostasis, cellular proliferation, differentiation, and apoptosis, leading to PAD progression [17,18]. These mutations can affect key regulators of vascular function, including endothelial nitric oxide synthase (eNOS) [19], vascular endothelial growth factor (VEGF) [20], and components of the renin-angiotensin system [21], contributing to the development and progression of PAD. Large-scale chromosomal rearrangements, including copy number variations and chromosomal translocations, can occur as somatic mutations in PAD, disrupting critical genes and regulatory regions and resulting in dysregulation of cellular processes involved in vascular biology, such as angiogenesis, ECM remodeling, and VSMCs function [22,23]. Denny et al. [24] utilized electronic health record (EHR) definitions of diseases to investigate the connection between CHIP and various types of atherosclerotic diseases affecting different vascular beds, including the mesenteric, coronary, cerebral, and aneurysmal vessels. The study found significant associations between CHIP and coronary artery disease (CAD), any aortic aneurysm, and chronic mesenteric ischemia. Notably, these associations were consistently more pronounced when large CHIP clones were present. Furthermore, CHIP was linked to the development of pan-arterial atherosclerosis, and once again, the effects were stronger in the presence of large CHIP clones. Of note, Zekavat et al. [25] classified the assessments of CHIP-PAD and CHIP pan-arterial atherosclerosis based on potential driver genes and certain mutations, centering on DNMT3A, TET2, ASXL1, JAK2, DDR gene, and mutations that disturb splicing-related factors. Their research revealed a connection between CHIP and peripheral artery disease (PAD), particularly involving the four frequently observed CHIP genes (DNMT3A, TET2, ASXL1, and JAK2). Importantly, they noted drastic heterogeneity in the effect sizes of PAD across these CHIP-related genes. Moreover, these findings disclosed the new outcome suggesting that TP53/PPM1D-CHIP relates to an occurrence of PAD/CAD, with a greater effect on PADs conferred by TP53. Among the mutations associated with CHIP, the role of TET2 is well-established in vascular disease. TET2’s normal function plays a significant role in regulating important processes in both macro- and microcirculation [26]. These processes include preventing the transformation of VSMCs, offering protection to ECs, and exerting anti-inflammatory and anti-atherogenic effects [27,28].

Maintenance of genomic integrity is crucial for proper vascular function, and impairments in DNA damage response can contribute to the accumulation of somatic mutations in PADs [29]. The intricate DNA repair pathways of cells counteract DNA damage and maintain genomic stability [30]. Chronic exposure to risk factors, like oxidative stress and chronic inflammation, can overwhelm the DNA repair capacity, leading to increased DNA damage and the accumulation of somatic mutations [31,32]. Defects in specific DNA repair genes, such as those involved in the BER or NER pathways, can further exacerbate DNA damage accumulation in PAD. Dysfunctional DNA repair and response pathways can perpetuate the accumulation of somatic mutations, promote genomic instability, and contribute to the progression of PAD [25,29]. In this respect, Zekavat et al. identified 338 and 419 PAD cases in the UK biobank (UKB) and Mass General Brigham Biobank (MGBB), respectively. Based on their results, CHIP was positively related to a high risk of PAD incidents in the UKB and MGBB [25]. More interestingly, Bick et al. revealed that those with larger CHIP clone sizes had a greater risk for PADs, which is associated more strongly with unfavorable clinical manifestations [33]. Specific genes affected by somatic mutations in PADs have been identified, and understanding their functional consequences is critical for unraveling disease mechanisms [2].

## 3. Genetic Landscape of Peripheral Arterial Diseases

Over the past decade, the prevalence of high throughput and affordable genotyping has led to genome-wide association studies (GWAS) becoming the predominant method for investigating the genetics of PADs. In 2019, Klarin and colleagues [13] conducted the largest GWAS study on PADs. They utilized EHR data and significantly increased the sample size by nearly 10 times through the analysis of the genetic biorepositories of the Million Veteran Program (MVP) and the UKB. The researchers examined roughly 32 × 10^6^ DNA variants for their relation to PADs. As a result, they identified 19 genomic regions that were significantly associated with PADs, 18 of them had not been reported previously. These genomic regions included genes, such as CELSR2/SORT1, F5, LPA, HLA-B, HDAC9, IL-6, LPL, ABO, CDKN2B-AS1/9p21, TCF7L2, MMP3, CREB3L1, PTPN11, RP11-359M6.3, COL4A1, SMOC1, CHRNA3, LOC732538, and LDLR. Further analysis revealed that the identified genetic variants were also associated with several known risk factors for PAD, including abnormal lipid levels, diabetic condition, smoking, thrombosis, and hypertension. Additionally, the researchers examined the impact of these genetic variants on atherosclerosis in peripheral, coronary, and cerebral arteries and observed that 14 of the risk variants for PADs displayed at least an insignificant relationship with CAD incidence, and 12 variants were linked to large artery stroke (LAS). Interestingly, when considering the presence of concomitant CAD or LAS, the effects of some genetic variants related to lipids (SORT1, LPA, and LDLR) on PAD risk were significantly reduced. This suggests that the shared causal pathways or comorbidities for atherosclerosis in different vascular beds may drive some of the risk for PADs. In contrast, PAD was distinctively linked to four significant variants found in the RP11-359M6.3, HLA-B, CHRNA3, and F5 (Leiden variant, p.R506Q) loci, while they were not associated with other types of atherosclerosis. Additionally, the COL4A1 locus, formerly linked to CAD [34] and cerebral microvascular disease [35], was related to both PAD and CAD. Overall, this study provides valuable insights into the genetic factors underlying PAD and their relationships with various risk factors and atherosclerosis. These mutations disrupt the balance of cell proliferation and apoptosis, contributing to the progression of vascular lesions and PAD pathogenesis. Understanding the specific molecular alterations induced by these mutations provides valuable insights into the dysregulated pathways and cellular processes driving disease progression in PADs.

Somatic mutations do not act in isolation but interact with other molecular alterations in PAD progression. The interplay between somatic mutations and factors, such as chronic inflammation, oxidative stress, and epigenetic modifications, can influence disease severity and outcomes [36]. For instance, somatic mutations can synergize with chronic inflammation to promote sustained immune activation and cytokine release, contributing to vascular damage and remodeling. In turn, the inflammatory microenvironment can further enhance the accumulation of somatic mutations through increased DNA damage and impaired repair processes [37]. Additionally, epigenetic modifications, such as DNA methylation and histone modifications, can modulate the impact of somatic mutations in PADs [38]. Aberrant epigenetic changes can affect gene expression patterns, including genes involved in vascular biology and DNA repair, amplifying the effects of somatic mutations on disease progression [39]. Understanding the intricate interplay between somatic mutations and other molecular alterations provides a comprehensive view of the complex mechanisms underlying PADs. Integration of these diverse factors is crucial for developing targeted therapeutic strategies that address the multifaceted nature of PADs and improve patient outcomes.

## 4. CHIP as Prognostic Markers in PADs

The studies revealed that somatic mutation accumulates in almost all of the tissues, and depending on the mutation variant, type, and gene region, it may cause disease. Zekavat et al. [25] demonstrated that PAD-CHIP carriers are characterized in older males who were previous smokers and have a history of CAD, hypertension, and hyperlipidemia, indicating a common vascular bed damage pathway as reported earlier [40,41]. Notably, larger CHIP clone sizes (as measured by VAF) have a significant strong effect on peripheral blood counts by increasing of pro-inflammatory total white blood cells, monocytes, neutrophils, and platelets than smaller CHIP clone sizes. For instance, a lack of TET2 in hematopoietic cells encourages abnormal macrophage production of inflammatory cytokines/chemokines, like IL-6 and IL-1. This establishes a direct connection between TET2 depletion and modifications to the BM niche’s microenvironment [42,43]. Furthermore, recent extensive cross-sectional research of MDS patients found that those with the mutations TET2, DNMT3A, and AXSL1 had a higher prevalence of PADs in the elderly population who were older than sixty [44]. According to above mentioned results, as well as recent elegant study, results disclosed that CHIP-carrier patients with PAD aged >65 were at a 3–4-fold higher risk of developing PAD than non-carriers. In another study, authors also demonstrated a graded association between CHIP VAF and PAD, as those with a VAF < 10% and a VAF > 10% increased the risk of a PAD incidence up to 58% and 100%, respectively [40]. These findings verify that CHIP is an independent risk factor that increases PAD events in a VAF-grade-dependent manner. Further studies are needed to elucidate the association between specific somatic mutations and clinical outcomes and the precise predictive value of somatic mutations in disease progression and recurrence.

### Accumulation of Mutant Clones in Ischemic Tissues of PAD Patients

The emerging evidence reveals a new insight into the infiltration of circulating mutant cells into ischemic tissue and the paravascular area, as well as their contribution to the development of atherosclerotic lesions. Büttner et al. [45] conducted a study sequencing 31 consecutive patients with PAD who underwent open surgical procedures, demonstrating that 45% of these patients had various CHIP gene mutations, including DNMT3A, TET2, ASXL1, and JAK2. Furthermore, almost 90% of the mutations detected in peripheral blood were also found in atherosclerotic lesions, perivascular fat, or subcutaneous tissues [45]. This data confirms that the circulating mutant clone infiltration into the ischemia tissues is also involved in the development of atherosclerosis in PAD patients. The contribution of the circulating CHIP-mutated clone to atherosclerosis lesion was also shown in the sample from carotid endarterectomy patients. The fraction of CHIP clones in circulating cells in peripheral blood correlated with the plaque fractions of the corresponding clones. In several cases, CHIP clones entering from the circulation contributed to more than 25% of the cell population in individual plaque segments [8]. In conclusion, somatic mutations play a significant role in the development of atherosclerosis in PAD patients. Accurate evaluation of the size of circulating cell somatic clones is necessary to predict atherosclerotic lesion progression. Future studies should focus on developing prognostic tools based on circulating cell somatic mutations to assess PAD development and management.

## 5. Diabetes Mellitus and Atherosclerosis as One of the Main Drivers of Somatic Mutation Accumulation in PAD Patients

An earlier investigation revealed a connection between type 2 diabetic mellitus (T2DM) and blood CHIP incidents [46]. There was a significant association between T2DM and CHIP incidents, especially in non-obese individuals who had T2DM. The association suggests that aging, long-term T2DM history, atherosclerosis, vessel formation, and revascularization may all play a role in mechanisms that lead to persistent glucotoxicity, oxidative stress, and inflammatory damage, which can then reduce the capacity of vascular cell lineages to regenerate [47,48]. Chronic inflammation and elevated reactive oxygen species can also prevent hematopoietic stem cells (HSCs) from self-renewing within the endosteal niche, which leads to ongoing premature mobilization of HSCs into the peripheral vessels and exhaustion of the reservoir of early myeloid progenitor cells with a pro-angiogenic secretory function [49,50]. These findings can be linked to the genetic profile of CH events in T2DM patients, which through the aforementioned processes prevents vascular regeneration and increases inflammation further. Compared to T2DM patients without clonal events, those with clonal mosaic events (71.4%) have a higher prevalence of vascular lesion, including microvascular and macrovascular diseases [46]. Heyde et al. [51] explored hematopoiesis in atherosclerotic animals and humans, and results demonstrated that the complex trait of atherosclerosis enhances HSC proliferation. Additionally, they determine whether mild hypercholesterolemia, in the absence of atherosclerosis, is sufficient to increase HSC proliferation and promote the expansion of mutant cells or not. The results indicate that mild hypercholesterolemia, without atherosclerosis, does not trigger increased HSC proliferation, nor does it lead to an altered expansion rate of Tet2^−/−^ myeloid cells. Taken together, multiple risk factors, such as T2DM-related impairments in stem/progenitor cell quality, atherosclerosis, metabolites, and hypercholesterinemia in the presence of atherosclerosis, significantly contribute to the accumulation of somatic mutations (Table 1).

## 6. Somatic Mutation Search in PAD-Related Thrombosis 

A recent meta-analysis has shown that CALR and JAK2V617F mutations are linked to alterations in blood counts and thrombosis [62]. JAK2V617F poses a high risk of thrombosis in the general population, even at and extremely low VAF, and is overrepresented among patients with thrombosis. The prevalence of JAK2V617F in each thrombosis group was higher than in the control group, indicating that the JAK2V617F mutation may increase the risk of both arterial and venous thrombosis, especially in the context of the population with clonal hematopoiesis. According to the subgroup analysis, the prevalence of the JAK2V617F mutation was highest in patients with splanchnic vein thrombosis (18.7%), then in those with ischemic stroke (8.5%), cerebral vein thrombosis (6.0%), deep vein thrombosis/pulmonary embolism (1.6%), and PADs (3.1%), respectively [62]. A previous report revealed that the prevalence of JAK2V617F mutation was higher in a cohort of patients with sonographically confirmed PADs compared to a group of healthy subjects. In comparison to healthy people, patients with PADs had a 5-fold greater frequency of JAK2V617F mutations. It is interesting to note that patients who took aspirin had a much lower frequency of mutations than those who did not [63]. Furthermore, it has been shown that JAK2 mutations are crucial for systemic inflammation, coagulation, reduced angiogenesis and proliferation, and thrombosis through the activation of the downstream STAT1,6-, MAPK-, and PI3K/AKT-signaling pathways [64]. Patients with the JAK2V617F mutation show altered blood endothelial cell outgrowth and increased expression of interferon-related genes, including chemokine ligand 2 and early growth response protein 1 as well as serine protease inhibitor B2 [65]. Patients with the JAK2V617F mutation’s platelets showed increased activation status and procoagulant potential. Additionally, JAK2V617F mutation carriers had a larger percentage of immature platelets than non-carriers, which can be more active than mature platelets [66]. Somatic mutation and thrombosis-related articles are comprehensively summarized elsewhere [67,68]. To sum, CHIP mutation or somatic mutation in PAD patients increases the risk of thrombosis significantly. Patients with PADs and an elevated or altered blood count need to be carefully screened to prevent thrombosis-related complications. Future studies are warranted to show the influence of antithrombotic therapy on the pathogenesis in patients with CHIP and somatic mutation.

## 7. Molecular Aspects of Somatic Mutation and Inflammation

While atherosclerosis is recognized as a major contributor to PAD pathogenesis, emerging evidence suggests that somatic mutations and associated inflammatory processes play a significant role in disease progression [12]. The inflammatory effects caused by specific mutations associated with CHIP have not been fully characterized; however, a shared characteristic seems to be the provocation of a state of increased inflammation. Individuals with evidence of CHIP exhibit elevated levels of pro-inflammatory factors, such as interleukin-6 (IL-6), tumor necrosis factor-α (TNF-α), and monocyte chemoattractant protein 1 (MCP-1), compared to those without CHIP [40,69]. It is believed that CHIP can trigger inflammation in immune cells that have genetic mutations via the rise in circulating inflammatory markers [69], as well as the presence of specific subsets of inflammatory cells [70]. Experiments conducted on BM cells containing TET2/DNMT3A mutations have further confirmed the heightened inflammatory capacity of CHIP [71]. Recent advancements in RNA sequencing of peripheral blood cells have further verified that the incidence of CHIP mutations induce an inflammatory profile in monocytes as well as T cells [72,73]. Analysis focusing on specific driver genes of CHIP revealed that TET2 mutations were related to overproduction of IL-1β, while JAK2 and SF3B1 mutations were linked to higher circulating levels of IL-18 [40]. As mentioned earlier, TET2, DNMT3A, JAK2V617F, and ASXL1 are the most frequently observed mutations causing CHIP, and the following is an overview of the underlying cascade resulting from CHIP with these mutations.

**TET2**: TET2 catalyzes the initial reaction in cytosine demethylation, a critical process for preserving the physiologic development of hematopoietic linage [74]. It also plays a crucial role in regulating the immunity, and reports indicate that TET2 mutation-driven CH contributes to the pathophysiology and advancement of vascular diseases by promoting the inflammation. Moreover, TET2 regulates the release of inflammation-related factors through the modulation of histone acetylation [42,75]. Studies on TET2-deficient mice have shown that stimulation of macrophages with lipopolysaccharide (LPS) and IFN-γ caused in overactivation of pro-inflammatory factors, like IL-1β and IL-6 [42]. Moreover, mice with TET2 deficiency in myeloid-derived cells exhibit increased expression of IL-1β via activation of the nucleotide-binding domain, leucine-rich-containing family, pyrin domain-containing-3 (NLRP3) inflammasome [26], IL-6 [75], and IL-8 [55]. In a cohort of subjects exclusive of vascular disease, the incidence of TET2 mutations was linked to a more than two-fold increase in levels of IL-8 when compared to individuals without these mutations [55].

**DNMT3A**: DNMT3A, the most commonly mutated gene in individuals with CHIP, regulates the transcription process by catalyzing DNA methylation. This gene also plays multiple roles in inflammation regulation. Specifically, it controls the expression of cytokines via controlling IQ motif-containing GTPase-activating protein 2 (IQGAP2), a scaffold protein of mast cells [76]. In individuals suffering from degenerative arthritis, the functionality of the IL-6 gene is associated with DNMT3A expression, and those with increased DNMT3A expression exhibit significantly lower levels of IL-6 secretion [77]. Additionally, in aortic valve stenosis, the incidence of mutations in DNMT3A is linked to a significantly higher ratio of TH17 to Tregs, indicating an inflammatory polarization of T cells [70].

**JAK2V617F**: Among the genetic abnormalities associated with CHIP, the JAK2V617F mutation is notably linked to pro-inflammatory conditions. This mutation acts as a downstream signal cascade for receptors of main cytokines, leading to the activation of inflammatory granulocytes and the formation of neutrophil traps [78]. These mutations are frequently found in myeloproliferative malignancies, like essential thrombocythemia and polycythemia vera [79], which are related to a higher risk of ischemic heart/brain attack and venous thromboembolism due to elevated blood viscidity and a coagulatory condition. Nevertheless, these mutations are progressively being detected in people with a normal peripheral blood profile and continue to be linked to a heightened risk of vascular mortality [80,81] and an increased production of pro-inflammatory cytokines and chemokines. Moreover, somatic mutations in genes associated with NF-κB signaling have participated in PAD progression [82]. Somatic mutations in gene-encoding components of the NF-κB pathway can dysregulate its activity, resulting in sustained inflammation and vascular dysfunction. Within this framework, the discovery that a genetic variation, specifically the EE genotype polymorphism of ICAM-1, significantly amplifies the susceptibility to PAD, underscores the notion that a pro-inflammatory status contributes significantly to the development of PAD [83]. Additionally, Flex and colleagues [84] conducted a study involving 157 individuals with PADs and 206 control participants. They discovered genetic variations.

**ASXL1**: The impact of ASXL1 mutations in CHIP on inflammation is not fully understood. However, analyses have proposed that ASXL1 mutations may have a critical role in promoting a pro-inflammatory state. ASXL1 mutation is prevalent in atherosclerosis/chronic ischemic heart-failure-affected individuals [85]. However, the precise mechanisms underlying this heightened CV risk are not yet fully understood. ASXL1 is involved in regulating gene expression and chromatin structure, and its mutations have been related to abnormal inflammatory cascades. In particular, ASXL1 mutations have been linked to an overproduction of pro-inflammatory elements in certain contexts [86]. Further research is needed to elucidate the specific mechanisms by which ASXL1 mutations contribute to inflammation in CHIP.

Understanding the molecular mechanisms linking somatic mutations and inflammation in PAD is of paramount importance. Recent studies have identified specific somatic mutations in PAD patients that directly contribute to the activation of inflammatory pathways. For instance, mutations in genes encoding toll-like receptors (TLRs) have been observed in PAD patients [87]. These mutations can result in enhanced TLR signaling, leading to molecules, including intercellular adhesion molecule-1, IL-6, E-selectin, MCP-1, and MMP1/3, were individually linked to PAD [84]. Of note, these mutations can disrupt the delicate balance between pro-/anti-inflammatory signals, leading to prolonged inflammatory status and the promotion of atherosclerosis. One notable consequence of somatic mutations in PAD is the induction of inflammatory elements, like NF-κB and AP-1 [85]. Mutated genes can aberrantly activate these transcription factors, leading to the upregulation of inflammatory cytokines, adhesion molecules, and matrix metalloproteinases [88]. This dysregulated inflammatory response promotes recruition of leukocyte, VSMCs proliferation, and ECM remodeling, all of which contribute to the development and progression of atherosclerotic lesions in PADs [89]. Furthermore, somatic mutations can also affect other inflammatory signaling pathways, including JAK/STAT pathway. JAK/STAT mutations can result in enhanced signaling and dysregulated cytokine responses, further perpetuating the inflammatory milieu in PADs [90].

Given the intricate interplay between CHIP and inflammation in PADs, targeting inflammation has emerged as a promising therapeutic strategy. By specifically addressing the molecular aspects associated with somatic mutations and inflammation, novel therapeutic interventions can be developed to attenuate disease progression. One potential approach is to target the dysregulated inflammatory signaling pathways directly linked to somatic mutations. These targeted therapies aim to restore the balance concerning pro-/anti-inflammatory signals, mitigating the detrimental effects of somatic mutations on inflammation and disease progression. Another therapeutic avenue lies in the development of immunomodulatory agents that can regulate the overall inflammatory response in PADs. By targeting common downstream mediators of inflammation, such as cytokines or chemokines, these agents could alleviate inflammation.

## 8. Somatic Mutation and Regeneration: Unraveling the Connection

Precision medicine approaches have revolutionized the field of healthcare by tailoring treatment strategies based on individual genetic profiles [91]. In the context of PADs, targeting somatic mutations has emerged as a promising avenue for precision medicine interventions [92]. Somatic mutations can have a profound impact on tissue regeneration and angiogenesis in PADs. By identifying specific somatic mutations present in individual patients, clinicians can develop personalized treatment plans that directly target the underlying genetic abnormalities contributing to disease progression. In studies exploring particular genes as potential candidates, the case-control method has been used to investigate allele frequency disparities between individuals with/without PADs, revealing various biological pathways implicated in sclerotic plaque formation and PADs [93,94,95]. Furthermore, involvement of the Notch signaling pathway has been documented in PAD subjects, which plays a crucial role in regulating VSMCs and macrophages. Activation of this pathway, specifically through the Delta-like 4 ligands, was associated with gene expression related to “inflamed plaque”, contributing to the adverse progress of PADs [96]. The principal genome study on PADs involved the Candidate Gene Association Resource consortium, which examined approximately 50,000 gene variants in over 29,000 individuals [97]. In European Americans, two variants, rs2171209 in SYTL3 and rs290481 in TCF7L2, showed significant associations with the ankle-brachial index (ABI). However, these associations were not observed in African Americans. The significance of these two single-nucleotide polymorphisms (SNPs) for ABI in European Americans could not be reproduced in a subsequent study containing 13,524 Europeans and Americans of European ancestry. Overall, candidate gene studies have faced challenges, including the random sorting of candidate genes and the repeated failure of replicating the outcomes in other studies [13]. As an example, certain NURR1 variants (rs13428968 and rs12803) were linked to restenosis/re-occlusion rates following angioplasty in a particular population [98], but this association was not observed in another population, and there was no association with either PAD or adverse cardiovascular events [99]. Understanding the mutation profiles of individual patients can guide the selection of targeted therapeutic strategies in PADs. Advances in genomic sequencing technologies have facilitated the identification of specific mutations associated with impaired tissue healing and angiogenesis. Based on the identified mutation profiles, therapeutic interventions can be tailored to address the specific genetic aberrations. For instance, small molecule inhibitors or gene therapies targeting genes with somatic mutations can be employed to modulate the downstream signaling pathways involved in tissue regeneration and angiogenesis. These targeted therapies hold the potential to restore normal cellular functions, promote tissue repair, and improve outcomes in PAD patients with specific mutation profiles.

Genetic alterations occurring in key genes involved in angiogenic and regenerative processes can disrupt the normal cellular mechanisms required for efficient tissue repair. Somatic mutations affecting genes encoding growth factors, receptors, or signaling molecules involved in angiogenesis can impair the formation of new blood vessels, leading to inadequate tissue perfusion and compromised healing in PADs [100]. For instance, it has been documented that mutations in the JAK2 gene have a crucial function in various inflammatory and coagulatory processes, reduced cell proliferation, and impaired vasculogenesis, which are mediated by the activation of underlying signaling pathways including STAT1,6, MAPK, and PI3K/AKT [64]. Mechanistically, individuals with the JAK2V617F mutation exhibit decreased outgrowth of blood ECs and increased expression of interferon-related genes, such as SERPIN-B2, EGR1, and chemokine ligand 2 [65], suggesting that individuals with the JAK2V617F mutation experience persistent inflammation and increased penetrability, a decreased cell proliferation rate (or vasculogenesis), and accelerated aging of EPCs compared with healthy individuals [65]. Investigating the influence of CHIP on tissue regeneration processes in PADs is a crucial area of research. By studying the functional consequences of specific mutations, researchers can uncover the molecular mechanisms by which genetic alterations impact tissue regeneration. Experimental models and in vitro studies can elucidate the effects of somatic mutations on cellular activities, like vasculogenesis, proliferation, recruitment, and ECM remodeling. These investigations can provide valuable insights into the specific pathways and molecular targets affected by somatic mutations, aiding in the development of novel therapeutic strategies aimed at enhancing tissue regeneration in PAD patients.

## 9. Conclusions

First, somatic mutation of multiple genes affects hematopoiesis, and the mutant clone size expands in circulating blood; the latter infiltrates into the ischemic tissue/atherosclerosis plaque and produces a variety of inflammatory cytokines, which contribute to the risk factors in the development of cardiovascular diseases, including PADs. Second, there is solid experimental evidence that hypercholesterinemia enhances HSC proliferation and leads to the chronic HSC proliferation process, which also significantly increases the inflammatory-thrombosis risk in patients with PADs. The patients with blood count abnormalities or elevated pro-inflammatory cytokines or interleukins and the elderly should be screened for further exposure to various risk factors that promote CHIP mutation, such as hypercholesterinemia, smoking, atherosclerosis, diabetes, and hypertension, so they can receive adequate care. Third, not all the mutated CHIP genes promote synthesis of inflammation-related factors, like IL-1β and IL-6. For instance, TP53 and PPM1D gene mutation does not produce a pro-inflammatory cytokine [40,41], while in patients with epigenetic genes (DNMT3A, TET2, and ASXL1), CHIP mutation circulating levels of pro-inflammatory cytokines are significantly increased [55,101,102]. When manipulating protective care approaches to address the impact of CHIP on atherosclerosis, it is essential to consider the mechanistic distinctions between TP53/PPM1D-CHIP and DNMT3A/TET2/ASXL1/JAK2-CHIP. For example, targeting IL-1b-driven inflammation, as demonstrated in the CANTOS analysis, could potentially prevent the pathogenic effects of TET2-CHIP [103]. The large VAF somatic-mutated TP53 genes in PAD development and prognosis were recently experimentally demonstrated in large group of more than 50,000 patient’s exome sequence data [25]. Further future studies require the establishment of a PAD patients’ disease prognostic calculator based on somatic mutated genes type, variant, and VAF grade, etc., to boost personalized therapy.

## Figures and Tables

**Table 1:** Peripheral artery disease and somatic mutations; Shared points and similarities.

PAD	Shared Points	Somatic Mutation
The risk of PADs markedly increases with age. Prevalence of PADs among individuals aged 80–100 years is 22 to 33% [52]	**Age**	Somatic mutations generally increase by around 2–6 mutations per cell division. Somatic mutation incidence increases in the ageing population by 10 to 20% at the age of 70 years [41,53].
Atherosclerosis incidence among PAD cases is more than 90% [54]	**Atherosclerosis**	Atherosclerosis patients are sufficient to produce a 3.5-fold increased risk of clonal hematopoiesis by age 70 [51]. Presence of somatic mutation in *TET2* increases atherosclerotic lesions of vessels by 3- to 4-fold [26,55].
Diabetic patients have 2- to 4-fold increased risk of developing PADs, CAD, and ischemic stroke [54]	**Diabetes mellitus**	Clonal mosaic event carriers with T2DM were associated with a 2-fold increase in the prevalence of vascular complications [46,56].
Approximately 70–80% of PAD patients exhibited elevated levels of inflammatory markers, suggesting a high incidence of inflammation in this population [57].	**Inflammation**	Inflammation, by generating reactive oxygen and nitrogen species that damage DNA, contributes to mutagenesis and can result in a 2- to 4-fold higher accumulation of somatic mutations compared to non-inflammatory conditions [32].
15–20% of PAD patients experienced a thrombotic event over a five-year follow-up period [58].	**Thrombosis**	The presence of JAK2 V617F mutation in PAD patients increased the risk of thrombosis by 3.1% [59].
Smoking is the most common risk factor for PAD occurrence with a population attributable fraction of 44% [54,60].	**Tobacco smoking**	Tobacco smoking dramatically increase the occurrence of somatic mutation from 1000 to 10,000 mutations per cell [61].

## Data Availability

Not applicable.
